# Print Materials to Promote Physical Activities in Japan: Content Analysis from a Goal Theory

**DOI:** 10.3390/healthcare11020239

**Published:** 2023-01-12

**Authors:** Tomomi Nagasawa, Tsuyoshi Okuhara, Marina Terada, Hiroko Okada, Eiko Goto, Takahiro Kiuchi

**Affiliations:** Department of Health Communication, School of Public Health, Graduate School of Medicine, The University of Tokyo, Tokyo 113-8655, Japan

**Keywords:** physical activity, goals, motivation, health communication, health promotion

## Abstract

Physical activity has significant health benefits for the heart, body, and mind. However, the percentage of people engaging in exercise routines is low in Japan. Goals are important components of motivation. Scholars suggest that appropriately setting both subordinate goals of what to do and superordinate goals of why to do it may motivate the audience and promote behavior. However, it is not known what goals are presented in print materials that promote physical activity. Therefore, this study aimed to understand the presented goals by performing content analysis of those materials in Japan. We collected print materials such as leaflets, brochures, and posters via website search. The presence of subordinate and superordinate goals and topics for each goal was analyzed. A total of 224 print materials were systematically collected and analyzed. The results showed that 14.3% of the print materials did not present any superordinate goals, whereas 100% of them presented subordinate goals. For superordinate goals, healthy aging was frequently presented. For subordinate goals, 67.4% presented only exercise. There is a difference in presenting goals between the private and government sectors. Since goals affect motivation and behavior change, it may be beneficial to incorporate the findings of the goal theory in future print materials.

## 1. Introduction

Physical activity has significant health benefits for the heart, body, and mind [1]. Physical activity is effective in the prevention and management of non-communicable diseases, such as cardiovascular disease, cancer, and diabetes, and reduces the symptoms of depression and anxiety [1,2]. People who are insufficiently physically active are reported to have a 20–30% higher risk of death, compared to those who are sufficiently active [1,2]. In Japan, it was estimated that 50,000 people died from physical inactivity in 2007, which is the third-highest risk factor after smoking and hypertension [3].

The World Health Organization (WHO) defines physical activity as any bodily movement produced by skeletal muscles that require energy expenditure and promotes physical activity to all people [1]. Physical activity includes not only exercise and sports but also work, mobility (e.g., walking), daily living activities, and household tasks [1,4]. The WHO announced guidelines for physical activity and sedentary behavior in 2020 and recommended having at least 150–300 min of moderate-intensity aerobic physical activity or at least 75–150 min of vigorous-intensity aerobic physical activity for adults aged 18 to 64 years or their equivalent combination [2]. In Japan, the Ministry of Health, Labour and Welfare (MHLW) promotes physical activity in the “Health Japan 21 (the second term),” which is a basic health promotion policy for Japanese people, and sets the goals of physical activity, such as increasing the daily number of steps, increasing the percentage of individuals who regularly exercise, and increasing the number of local governments that offer community development and environments to promote physical activity [5]. 

However, only 35.9% of men and 28.6% of women have an exercise routine in Japan, and there has been no significant increase or decrease over the past 10 years [6]. The number of steps walked in daily life has been decreasing in recent years [6]. According to the online survey conducted by the Japan Sport Association in January 2022, approximately 40% of Japanese adults aged from 20 to 59 answered that they did not want to exercise [7]. Therefore, how to effectively motivate people needs to be addressed. 

People are considered to be motivated and act when they perceive value in their goals [8]. Goals are desired end states that people wish to approach or avoid [9]. Goals are a central concept in motivation [10] and activate, energize, and sustain behavior [11]. Goals include “subordinate goals” (e.g., walking 10,000 steps every day), which are specific and describe what we do, and “superordinate goals” (e.g., good quality of life), which are abstract and describe why we would like to do them [12]. In goal-setting theory, it is argued that making subordinate goals specific eliminates ambiguity about what needs to be achieved, thereby bringing them closer to the expected performance [11,13]. Goal-setting also increases self-efficacy [11,14]. Self-efficacy is an individual’s belief in their ability to succeed in executing tasks [15]. By setting specific goals, a sense of self-efficacy is fostered when achieving each goal [15], leading to even higher performance [11,13]. On the other hand, it has been discussed that subordinate goals have the potential to encourage short-term thinking and narrow one’s perspective to achieve the goals [16]. 

Abstract superordinate goals are positioned above subordinate goals [17]. Superordinate goals reflect one’s sense of self [17]. Subordinate goals are set to achieve superordinate goals; therefore, superordinate goals can promote the goal-seeking behavior of subordinate goals [17,18]. For example, exercising once a week is a subordinate goal of the superordinate goal of getting healthy, and the presentation of the superordinate goal of getting healthy may promote exercise once a week. The study indicates that setting both subordinate and superordinate goals promotes goal-seeking behavior, compared to setting only either goal [12]. In this study, people set their new-year resolution using four types of goal-setting methods (subordinate goals only, superordinate goals only, both subordinate goals and superordinate goals, and self-setting goals), and people who set both subordinate and superordinate goals have the most goal-seeking behavior after three months [12]. Therefore, not only setting subordinate goals but also setting superordinate goals may be an effective strategy for achieving long-term goals [9,12]. 

Several studies showed that the difference in superordinate goal categories affects performance. Segar (2011) measured the superordinate goal of physical activity among middle-aged women and investigated its relationship with participation levels in physical activity after 1 year [19]. Even though participants valued healthy-aging, current-health, and quality-of-life (QOL) equally, those with QOL goals participated in more physical activity than those with healthy-aging and current-health goals [19]. Another study investigated the effects of advertisements that framed physical activity as a way to be healthier, lose weight, or enhance daily well-being and reported that framing physical activity as a way to enhance daily well-being had a positive impact on perceptions of their physical activity experiences and body image in overweight women [20]. It has been argued that QOL goals are autonomous and intrinsic motivators, whereas health and weight goals are extrinsic motivators that reflect social norms [19,20]. Although the goal of health seems autonomous, it may reflect social norms that are desirable in culture, thus making exercise a moral imperative [21,22]. Another factor as to why QOL goals were more effective than others might be the influence of the reward and its timing. Since people tend to choose small, immediate rewards over larger rewards that occur over time [20,23], it is argued that physical activity might be promoted by immediate benefits such as exhilaration. Although these studies cannot be generalized due to the limited study population of middle-aged women in the United States, they present an interesting insight into QOL goals that may be more effective than goals commonly found in physical activity such as health and weight loss. These studies indicate that the influence of superordinate goals on physical activity may depend on the categories of superordinate goals.

Goals are activated either consciously or unconsciously [8]. Goal priming is the activation of goals by external cues and influences goal-pursuit behavior toward the primed goal [24]. The meta-analysis showed that exposure to a goal-related word triggered a goal-directed behavior, and goal priming was particularly effective when the primed goal was perceived as important to the audience [25]. Therefore, it is critical to present goals that the audience highly values. 

Since goal priming is a nonconscious motivational strategy [24], passive information touchpoints such as posters, leaflets, news, and advertisements could be implementation fields for goal priming. Print materials (e.g., leaflets, brochures, and posters) are the most practical way to provide information and, in some cases, print materials may be more effective in promoting physical activity than web-based messages [26,27]. However, few studies have examined the content of print materials that promote physical activity. A study of content analysis of walking brochures in the United Kingdom reported that the most frequently presented contents were walking directions and landscape, and there were few messages about the health benefits of physical activity and encouraging behavior change [28]. The other content analysis of brochures in the United States and Canada examined the messages based on behavior change theory and reported that <30% of the brochures contained messages on self-efficacy, outcome expectations, and self-regulation, which were considered promising for promoting physical activity [29]. As noted, studies on the content of print materials that promote physical activity are limited, and no study has utilized goal theory for the analysis.

Goals are an important component of motivation, and effective goal-setting increases motivation and promotes behavioral change. Therefore, it is important to understand what goals are being presented in print materials to promote physical activity. This study aims to understand the goals presented in print materials that promote physical activity by performing content analysis. In particular, we are interested in the presence of superordinate goals of “why to do”, since they could be lacking while the subordinate goal of “what to do” is must that should be included in the content. Specifically, this study aims to examine the following four research questions: (1) what percentage of print materials that promote physical activity present superordinate goals; (2) what categories and topics of superordinate goals are presented; (3) what categories and topics of subordinate goals are presented; and (4) what are the differences between publishers? 

## 2. Materials and Methods

### 2.1. Design

We performed content analysis of the physical activity (PA) print materials in Japan. We examined qualitative data of the text presented in the PA print materials and quantitatively analyzed the presented goals’ categories and topics.

### 2.2. Material Collection

We collected print materials of leaflets, brochures, and posters to promote physical activity targeting adults over 18 years of age, which are distributed by the Japanese government, local governments, and health insurance societies. Since most people may not be motivated to exercise in Japan, it is assumed that they do not seek information on physical activity proactively. Therefore, passive information such as leaflets, brochures, and posters from insurers such as local governments and health insurance societies were targeted in this study, rather than active information such as website articles obtained through online searches. Specifically, website articles were not eligible for this study, but the PDFs of leaflets and posters located on the website were eligible because they could also be distributed offline. 

The PA print materials were collected in three systematic ways via website search in July 2022. First, we searched the websites of the MHLW [30,31,32] and Japan Sports Agency [33]. The MHLW and the Japan Sports Agency are the major government organizations promoting physical activity in Japan; therefore, we collected PDFs of PA print materials on their websites. Second, we used the two most popular search engines in Japan (Google Japan and Yahoo! Japan) to extract the PA print materials distributed by local governments and health insurance societies. Google Japan and Yahoo! Japan account for 76.59% and 14.24% of Japanese searches, respectively [34]. The search was performed using the keywords “physical activity” OR “exercise”. Although physical activity is a concept that includes exercise, the physical activity guideline from the MHLW uses both physical activity and exercise [35]. Therefore, we used both physical activity and exercise as our search terms. The top 100 webpages were extracted from each search engine. After eliminating duplicates, websites that did not reach the PDFs of the PA print materials within two clicks of the found page were excluded. We also excluded websites that included PDFs that did not provide information on physical activity for the general public (e.g., policy reports), targeted non-adults (e.g., school programs), targeted people with specific diseases (e.g., people living with diabetes), catered for special situations such as evacuation living, and walking maps that did not mention physical activity. Third, we collected PA print materials purchased by local governments and health insurance societies from private publishers for distribution to the audience. Publishers were identified by a Google search with the keywords “specific health guidance” AND “publisher”. In Japan, the implementation of specific health guidance to insured persons is mandated by insurers [36]; therefore, publishers developing print materials for specific health guidance are considered to be developing PA print materials for insurers as well. The PA print materials were selected from online catalogs on each company’s website. We contacted the identified companies, confirmed that the selected materials were used by insurers, and asked them to provide samples. When samples were not available, they were purchased. Material collection and selection were conducted by the first author (TN), consulting with the second author (TO) when necessary.

### 2.3. Coding Procedure

The presence of subordinate and superordinate goals and topics for each goal were coded. The unit of coding was each material, and the presence of each item in the materials was coded as 1 in a Microsoft Excel coding sheet developed by TN. Material publishers (government, private, or other), material format (leaflets or posters), material size, and material volume were recorded. We defined leaflets as the print materials intended for distribution, with a wide range from one-page leaflets to multi-page booklets.

The superordinate goals were classified into four categories according to a previous study [19]: Healthy-aging, Current-health, Appearance/weight, and Quality-of-Life. Healthy-aging is a goal related to healthy life expectancy and future health; current-health is a goal focused on current health conditions, such as blood pressure reduction and healthy lifestyle; appearance/weight is a goal related to weight loss and obesity reduction; quality-of-life is a goal that includes stress reduction and better sleep. Healthy-aging and current-health are both health-related goals, but healthy-aging is future-focused and includes disease prevention. When coding, we had the Others category, but no materials were categorized in Others; therefore, we only used the four categories from the previous study. We also counted the number of superordinate goal categories presented for each material, which ranged from 0 to 4. The subordinate goals were classified into two categories: exercise and daily life activities, following the classification of the physical activity guidelines by the MHLW [35]. 

For both superordinate and subordinate goals, specific goal descriptions in the materials were extracted into the coding sheet to understand the topics presented under each goal category. By reviewing the extracted descriptions of all print materials, TN inductively created topics for each category and developed coding guidelines. In this study, we focused on the presence of goal categories and topics; therefore, we did not count the number or location of goals presented in the print materials. 

### 2.4. Analysis

TN provided a one-hour briefing session on using coding guidelines (Appendix A) to the third author (MT). In the briefing session, TN and MT conducted a pilot coding using ten randomly selected articles. When there were differences between TN’s coding and MT’s coding, TN and MT discussed these until a consensus was reached and the coding guideline was improved. Finally, 25% of the final dataset (56/224) was randomly selected and evaluated by MT independently to ensure inter-rater reliability. Data were analyzed using Statistical Package for the Social Sciences version 21.0 (SPSS, Chicago, IL, USA). Inter-rater reliability was assessed using Cohen’s kappa for the coding of the goal category and the number of superordinate goal categories in a material. A guideline by Landis and Koch (1977) provided the basis for the interpretation of reliability estimates as follows: 0 to 0.20, slight agreement; 0.21 to 0.40, fair agreement; 0.41 to 0.60, moderate agreement; 0.61 to 0.80, substantial agreement; and greater than 0.80, almost perfect agreement [37]. 

Chi-square analysis or Fisher’s exact test was used to analyze differences between the number of superordinate goals and the category of superordinate and subordinate goals.

## 3. Results

### 3.1. Inter-Rater Reliability

The inter-rater reliability of the two reviewers was high. Total agreement and the Cohen’s kappa coefficient for each goal category were as follows: 98.2% and 0.964 for healthy-aging; 98.2% and 0.962 for current-health; 98.2% and 0.962 for appearance/weight; 98.2% and 0.941 for quality-of-life; 100% and 1.000 for exercise; and 94.5% and 0.874 for daily life activities. For the number of superordinate goal categories in a material, the total agreement was 94.6% and the Cohen’s kappa coefficient was 0.917. We used the coding of the first author for the data analysis in this study. 

### 3.2. Basic Characteristics

A total of 224 PA print materials were collected. Table 1 presents the sample characteristics. Of the 224 PA print materials, 64.7% were materials developed by the private sector (private materials), 31.7% were materials developed by the government (government materials), while 3.6% were materials developed by others (other materials), such as academic societies and health promotion organizations. The majority (86.2%) were leaflets and 13.8% were posters. With regard to material size, 51.8% were A4 size and 28.6% were in the free category, which could be resized according to the printing specifications. The government and other materials that did not mention size restrictions were categorized as the free category. A total of 57.1% had 1–2 pages of material volume.

### 3.3. Numbers of Superordinate Goals in the PA Print Materials

Among all materials, 14.3% did not present any superordinate goals. Table 2 shows that 41.1% of the materials presented a single superordinate goal and 44.6% presented two or more superordinate goals. There is a significant difference between publishers in the number of goals presented in the PA print materials. 

### 3.4. Categories and Topics of Superordinate Goals in the PA Print Materials

Table 3 lists the categories of superordinate goals presented in the materials. Among the 193 materials that presented at least one superordinate goal, healthy-aging was the most common category of superordinate goal (69.4%), followed by appearance/weight (47.2%), current-health (45.1%), and quality-of-life (32.6%). There is a significant difference between publishers in the percentage of presented categories of healthy-aging, appearance/weight, and quality-of-life. We also analyzed the topics under each superordinate goal category, as shown in Table 3. The most common topic presented in healthy-aging was the prevention of lifestyle-related diseases (59.7%), followed by healthy life expectancy (38.8%) and the prevention of locomotive syndrome (32.1%). For the topics in other superordinate goal categories, functionality improvement was presented in 34.5% under current-health, obesity/fat reduction was presented in 59.3% under appearance/weight, and mental health/stress reduction was presented in 93.7% under quality-of-life.

### 3.5. Categories and Topics of Subordinate Goals in the PA Materials

Among all materials, 100% presented at least a single subordinate goal. Table 4 shows the categories of subordinate goals in the materials. Among these categories, 67.4% presented only exercise, 2.2% presented only daily life activities, and 30.4% presented both exercise and daily life activities. Of the poster materials, 12.9% presented daily life activities only, which resulted from several posters promoting stair use. All publishers presented exercise the most, and private materials had the highest percentage of both exercise and daily life activities (40.7%) among all publishers, which showed a significant difference.

Table 5 shows topics under each subordinate goal category. Walking was presented most frequently in exercise (53.9%), followed by strength training (45.2%) and stretching (42.5%). While walking was most frequently mentioned in the private and the other materials (65.5% and 57.1%, respectively), local calisthenics (34.3%) was most frequently presented in the government materials. Local calisthenics is an exercise developed independently by local governments. In terms of daily living activities, daily life mobility, stair use, and household tasks were all promoted at the same level, at more than 70%. 

## 4. Discussion

Regarding the RQ1 (i.e., number of superordinate goals), this study showed that some materials did not present any superordinate goals, and 41.1% of the materials presented only a single superordinate goal. As mentioned earlier, the goal theory suggests that presenting both superordinate and subordinate goals and priming goals that are highly valued by individuals promote goal-directed behavior [12,25]. Goal pursuit is assumed to be resource-dependent [38]. The presence of multiple goals linked to a given means defines the multifinality set, known as “many birds with one stone”, and this has proven to play an important role in means preferences [18,39]. For example, when a prize package is framed as fitness alone, entertainment alone, or both fitness and entertainment, the presentation of two or more goals results in a higher intention to participate in the lottery than a single presentation [18]. This suggests that “many birds with one stone” is an important determinant of preference and that presenting multiple goals may be more effective than single goals. Based on these theoretical findings, this study result indicates that current PA print materials may not be effectively promoting physical activity to the audience. In future materials, it may be beneficial to present multiple superordinate goals.

With regard to RQ2 (i.e., categories and topics of superordinate goals), our study result about the category of superordinate goals presented in PA print materials is consistent with those of the previous study [19]. The result showed that healthy-aging was the most common superordinate goal category, followed by appearance/weight, current-health, and quality-of-life. With healthy-aging, prevention of lifestyle-related diseases and healthy life expectancy were presented the most. It is assumed that the high prevalence of the healthy-aging category is influenced by government policies. In 2000, Japan’s MHLW set the goal of extending healthy life expectancy as a part of Health Japan 21 and introduced specific health checkups and specific health guidance, focusing on metabolic syndrome for the primary prevention of lifestyle-related diseases [5]. As it is assumed that PA print materials will be used in specified health checkups and specified health guidance, the materials may reflect the policy intention. Appearance/weight was the second highest, which might have resulted from the influence of the media. Media are known to have a strong influence on the formation of an ideal body image [40,41]. After 1950, many skinny models began to appear in the media, and it was emphasized that being thin is an element of external beauty [42]. PA print materials might reflect the values created by the media. However, healthy-aging and appearance/weight goals may not always be effective in promoting physical activity. As mentioned earlier, a previous study indicated that QOL goals are more effective than health and appearance goals due to their autonomy and immediacy of the rewards [19]. In future materials, it may be beneficial to present intrinsic goals, such as improving QOL, rather than extrinsic goals that reflect social norms such as health and appearance.

With regard to RQ3 (i.e., categories and topics of subordinate goals), this study showed that 67.4% promoted only exercise, which may not be an effective strategy to promote physical activity. Negative attitudes toward exercise inhibit physical activity [43]. Several meta-analyses have shown that a lack of interest or motivation in physical activity, dislike of exercise, and lack of time have been reported as inhibitors of exercise, and self-efficacy has been reported as a promoter of exercise [44,45,46]. WHO emphasizes “every move counts,” and promotes any movement, including daily life mobility and household tasks as physical activity in the guidelines of physical activity and sedentary behavior [2]. The new guidelines lower the hurdles of physical activity and encourage people to move, regardless of the type and duration of activity, which would change the perception toward physical activity and increase self-efficacy. The goal theory suggests that increasing self-efficacy toward goals improves performance [11,14,15]. Since daily life activities are easier to work on for people who do not have time, these may increase their self-efficacy. In future materials, it may be beneficial to present both exercise and daily life activities.

With regard to RQ4 (i.e., the difference between publishers), this study showed several differences in the superordinate and subordinate goals between publishers. It was noted that 35.2% of the government materials did not have a superordinate goal compared to 4.8% of the private materials. Furthermore, while 40.7% of the private materials promoted both exercise and daily life activities, only 14.1% of the government materials did so. Based on the findings of previous studies mentioned earlier, the results indicate that private materials may be more effective than government materials in promoting physical activity. The quality differences among publishers may be a result of competition. While government materials are developed by the government and are directly distributed to the audience, private materials are developed by private publishers, purchased by the government and health insurance societies, and delivered to the audience. In other words, private materials need to survive selection competition, which may lead to quality improvement of the materials. 

Differences between government and private materials were also found in the types of exercises promoted. Walking was the most frequently promoted exercise in private materials (65.5%), whereas local calisthenics was the most promoted in government materials (34.3%). Local calisthenics is an exercise created by local governments to promote physical activity among residents by incorporating famous local places, festivals, specialties, and dialects into movements and lyrics. As of 2018, approximately 700 such exercises have been identified, accounting for more than one-third of the local governments [47]. While local calisthenics is expected to be effective in making exercise more familiar and promoting physical activities, awareness of them tends to be low [47]. A study of people aged over 65 years old in rural areas showed that 93.2% of men and 63.6% of women had never practiced local calisthenics [48]. Local calisthenics has been expanding since the local government formulated local health promotion plans, which are a component of Health Japan 21 [47]. Local calisthenics is not born out of the audiences’ needs but out of the policy needs of local governments. The inclusion of local calisthenics in the materials may be effective in achieving policy goals, but they may not be effective in achieving the essential goal of increasing the audience’s physical activity.

Based on the findings of the goal theory, this study result indicates that PA print materials currently in use have room for improvement, especially for the materials developed by the government. Since public health practitioners in the local government have multiple roles and there is no health communication specialist in their organizations, it might be difficult for them to create effective health promotion materials from scratch. The findings of the goal theory, such as presenting both superordinate and subordinate goals and highly valued goals such as QOL goals, would be good tips for public health practitioners, especially developers and disseminators of health communication messages, to incorporate in their communication materials.

This study has several limitations. First, the materials covered in this study do not reflect all the physical activity materials currently in use by the government and health insurance societies in Japan. Although we collected government materials from websites, some government materials may not have been available online. The second limitation concerns the search keywords. Since physical activity includes a variety of sports and exercises, we may miss the materials specific to them. Third, this study did not cover materials from other organizations, such as private fitness clubs. Fourth, although we developed a detailed coding guideline to conduct systematic analysis and coding, our results may reflect a coder bias. Fifth, because this study only included leaflets and posters in Japanese, generalization to other contexts is limited. Sixth, we did not count the frequency and location of goals presented in the materials, which may have influenced audience recognition of goals. Seventh, we did not separately analyze the PA print materials by age and sex of targeted audiences because only few materials targeted a specific sex or age. Finally, since this study is limited to a content analysis of PA print materials, it is unknown how these materials affect audience awareness and behavior, and it should be investigated in future studies. Despite these limitations, to the best of our knowledge, this is the first study to identify the goals (superordinate and subordinate goals) presented in PA print materials.

## 5. Conclusions

This study aimed to understand the goal presented in PA print materials by conducting content analysis. This study showed that approximately 15% of the materials did not present any superordinate goals, whereas 100% of them presented subordinate goals. The superordinate goals frequently presented in the materials were healthy-aging, and more than half of the materials presented only exercise as a subordinate goal. There is a difference between the private and government sectors in presenting superordinate and subordinate goals in the materials. Since goals affect motivation and behavior change, it may be beneficial to incorporate the findings of goal theory in future print materials, such as presenting both superordinate and subordinate goals and referring to QOL goals.

## Figures and Tables

**Table 1:** Sample characteristics (*N* = 224).

Characteristics		N	%
Publisher	Government	71	31.7%
	Private	145	64.7%
	Others	8	3.6%
Material form	Leaflet	193	86.2%
	Poster	31	13.8%
Material size	A1	19	8.5%
	A2	6	2.7%
	A3	4	1.8%
	A4	116	51.8%
	A5	5	2.2%
	B5	4	1.8%
	B6	5	2.2%
	B7	1	0.4%
	Free	64	28.6%
Material volume (page)	1	67	29.9%
	2	61	27.2%
	3–10	59	26.3%
	11–20	23	10.3%
	21–30	6	2.7%
	31–40	8	3.6%

**Table 2:** Number of Superordinate Goals in a PA Print Material (*N* = 224).

# of Goals	0	1	>2	*p*-Value
Total (*N* = 224)	32 (14.3%)	92 (41.1%)	100 (44.6%)	
Form ^a^				
- Leaflet (*N* = 193)	26 (13.5%)	77 (39.9%)	90 (46.6%)	0.308
- Poster (*N* = 31)	6 (19.4%)	15 (48.4%)	10 (32.3%)	
Publisher ^b^				
- Government (*N* = 71)	25(35.2%)	28 (39.4%)	18 (25.4%)	<0.001
- Private (*N* = 145)	7 (4.8%)	58 (40.0%)	100 (55.2%)	
- Others (*N* = 8)	0 (0.0%)	6 (75.0%)	2 (25.0%)	

^a^ Significant differences were determined by chi-square analysis. ^b^ Significant differences were determined by Fisher exact test.

**Table 3:** Categories and Topics of Superordinate Goals in PA Print Materials by Publisher (*N* = 193).

	Total (*N* = 193)		Government (*N* = 47)		Private (*N* = 138)		Others (*N* = 8)		*p*-Value
**Goal Category** Topic	**N**	**%**	**N**	**%**	**N**	**%**	**N**	**%**	
**Healthy-aging ^b^**	**134**	**69.4%**	**23**	**48.9%**	**105**	**76.1%**	**6**	**87.5%**	**<0.001**
Healthy life expectancy	52	38.8%	9	39.1%	42	40.0%	1	16.7%	
Prevention of lifestyle-related disease	80	59.7%	11	47.8%	67	63.8%	2	33.3%	
Pain prevention	15	11.2%	2	8.7%	12	11.4%	1	16.7%	
Osteoporosis prevention	11	8.2%	1	4.3%	10	9.5%	0	0.0%	
Dementia prevention	37	27.6%	8	34.8%	27	25.7%	2	33.3%	
Prevention of depression	18	13.4%	6	26.1%	12	11.4%	0	0.0%	
Prevention of other health problems	1	0.7%	0	0.0%	0	0.0%	1	16.7%	
Anti-aging	7	5.2%	1	4.3%	6	5.7%	0	0.0%	
Prevention of locomotive syndrome	43	32.1%	10	43.5%	30	28.6%	3	50.0%	
Prevention of frailty	10	7.5%	2	8.7%	7	6.7%	1	16.7%	
Prevention of falls/fracture	24	17.9%	4	17.4%	19	18.1%	1	16.7%	
Prevention of elderly care	24	17.9%	3	13.0%	20	19.0%	1	16.7%	
**Current-health ^b^**	**87**	**45.1%**	**30**	**63.8%**	**55**	**35.5%**	**3**	**37.5%**	**0.933**
Health-related value improvement	22	25.3%	2	6.7%	20	36.4%	0	0.0%	
Pain relief	20	23.0%	5	16.7%	14	25.5%	1	33.3%	
Functionality improvement	30	34.5%	6	20.0%	24	43.6%	0	0.0%	
Physical improvement	22	25.3%	4	13.3%	16	29.1%	2	66.7%	
Improvement of other health issues	7	8.0%	0	0.0%	7	12.7%	0	0.0%	
Health/Healthy lifestyle	21	24.1%	14	46.7%	7	12.7%	0	0.0%	
**Appearance/weight ^b^**	**91**	**47.2%**	**8**	**17.0%**	**83**	**59.4%**	**1**	**12.5%**	**<0.001**
Obesity/fat improvement	54	59.3%	2	25.0%	51	61.4%	1	100.0%	
Metabolic syndrome/visceral fat improvement	27	29.7%	4	50.0%	23	27.7%	0	0.0%	
Weight/body shape improvement	39	42.9%	3	37.5%	36	43.4%	0	0.0%	
**Quality-of-life ^a^**	**63**	**32.6%**	**13**	**27.7%**	**50**	**35.5%**	**0**	**0.0%**	**0.009**
Mental health/Stress reduction	59	93.7%	10	76.9%	49	98.0%	0	0.0%	
Better sleep/Recovery from fatigue	20	31.7%	4	30.8%	16	32.0%	0	0.0%	
Other QOL	1	1.6%	0	0.0%	1	2.0%	0	0.0%	

For category, the denominator in the category percentage is 193, which is the number of materials containing at least one superordinate goal. For topics, the denominator in the topic percentage is the number of materials in each category. ^a^ Significant differences were determined by chi-square analysis. ^b^ Significant differences were determined by Fisher exact test.

**Table 4:** Categories of Subordinate Goals in the PA Print Materials (*N* = 224).

Subordinate Goal Category	Exercise Only	Daily Life Activities Only	Both Exercise and Daily Life Activities	*p*-Value
Total (*N* = 224)	151 (67.4%)	5 (2.2%)	68 (30.4%)	
Form ^b^				
- Leaflet (*N* = 193)	129 (66.8%)	1 (0.5%)	63 (32.6%)	0.002
- Poster (*N* = 31)	20 (64.5%)	4 (12.9%)	7 (22.6%)	
Publisher ^b^				
- Government (*N* = 71)	57 (80.3%)	4 (5.6%)	10 (14.1%)	<0.001
- Private (*N* = 145)	86 (59.3%)	0 (0.0%)	59 (40.7%)	
- Others (*N* = 8)	6 (75.0%)	1 (12.5%)	1 (12.5%)	

^b^ Significant differences were determined by Fisher exact test.

**Table 5:** Categories and Topics of Subordinate Goals in the PA Print Materials by Publisher (*N* = 224).

	Total (*N* = 224)		Government (*N* = 71)		Private (*N* = 145)		Others (*N* = 8)		*p*-Value
**Goal Category** Topic	**N**	**%**	**N**	**%**	**N**	**%**	**N**	**%**	
**Exercise** ^b^	**219**		**67**		**145**		**7**		**0.002**
Walking	118	53.9%	19	28.4%	95	65.5%	4	57.1%	
Jogging/Running	33	15.1%	5	7.5%	27	18.6%	1	14.3%	
Cycling	16	7.3%	0	0.0%	16	11.0%	0	0.0%	
Swimming	32	14.6%	3	4.5%	28	19.3%	1	14.3%	
Other aerobic exercises	29	13.2%	5	7.5%	23	15.9%	1	14.3%	
Strength training	99	45.2%	18	26.9%	77	53.1%	4	57.1%	
Stretching	93	42.5%	21	31.3%	71	49.0%	1	14.3%	
Exercising while doing something	40	18.3%	5	7.5%	34	23.4%	1	14.3%	
Radio calisthenics	25	11.4%	3	4.5%	20	13.8%	2	28.6%	
Training for locomotive syndrome	9	4.1%	1	1.5%	6	4.1%	2	28.6%	
Local calisthenics	24	11.0%	23	34.3%	0	0.0%	0	0.0%	
Other exercises	57	26.0%	11	16.4%	44	30.3%	2	28.6%	
Exercising	19	8.7%	4	6.0%	15	10.3%	0	0.0%	
Sports	27	12.3%	4	6.0%	22	15.2%	1	14.3%	
**Daily life activities** ^a^	**75**		**14**		**59**		**2**		**0.008**
Daily life mobility	56	74.7%	6	42.9%	49	83.1%	1	50.0%	
Stair use	57	76.0%	10	71.4%	46	78.0%	1	50.0%	
Household tasks	53	70.7%	7	50.0%	45	76.3%	1	50.0%	
Moving frequently	26	34.7%	5	35.7%	20	33.9%	1	50.0%	
Leisure time life activity	40	53.3%	5	35.7%	35	59.3%	0	0.0%	

^a^ Significant differences were determined by chi-square analysis. ^b^ Significant differences were determined by Fisher exact test.

## Data Availability

Publicly available datasets were analyzed in this study. The data collected from the Internet can be found at https://www.google.com/ and https://www.yahoo.co.jp/ (accessed on 19 July 2022).

## Appendix A

**Table A1:** Coding items.

Coding Categories	Coding Items	Description
Publisher	Government	National and local governments
	Private	Private publishing companies
	Others	Academic societies, health-promoting organizations, etc.
Material form	Leaflet	Material intended for distribution, with a wide range from one-page leaflets to multi-page booklets
	Poster	Material named posters
Material size		Material size, such as B5, A4, A2, etc. Free is a category, which can be resized according to the printing specifications
Material volume		Number of pages of a print material
Category of superordinate goals	Healthy-aging	Goals related to healthy life expectancy and future health including disease prevention
	Current-health	Goals focused on current health conditions, such as blood pressure reduction and a healthy lifestyle
	Appearance/weight	Goals related to weight loss and obesity reduction
	Quality-of-life	Goals related to QOL such as stress reduction and better sleep
Topics of healthy-aging	Healthy life expectancy	Reference to healthy life expectancy and healthy longevity
	Prevention of lifestyle-related disease	Reference to the prevention of lifestyle-related diseases such as cancer, cardiovascular disease, diabetes, and COPD
	Pain prevention	Reference to the prevention of physical pain such as back pain and stiff shoulders
	Osteoporosis prevention	Reference to the prevention of osteoporosis
	Dementia prevention	Reference to the prevention of dementia and mild cognitive impairment
	Prevention of depression	Reference to the prevention of depression
	Prevention of other health problems	Reference to other health issues other than those listed above, such as economy class syndrome and lifestyle inactivity diseases
	Anti-aging	Reference to anti-aging
	Prevention of locomotive syndrome	Reference to the prevention of locomotive syndrome, sarcopenia, and declining legs
	Prevention of frailty	Reference to the prevention of frailty
	Prevention of falls/fracture	Reference to the prevention of falls, injuries, and fractures
	Prevention of elderly care	Reference to independent living and the prevention of elderly care
Topics of current-health	Health-related value improvement	References to improvement in blood pressure, hyperlipidemia, blood glucose level, or to improvement in pre-existing conditions
	Pain relief	Reference to the relief of physical pain, such as stiff shoulders, back pain, and headaches
	Functionality improvement	Reference to functionality improvements, such as improved cardiopulmonary function, improved brain function, and improved immunity
	Physical improvement	Reference to maintaining or improving physical fitness and muscle strength
	Improvement of other health issues	Reference to improving other health problems, such as sensitivity to colds, constipation, and chronic headaches
	Health/Healthy lifestyle	Reference to general health, such as getting healthy, living a healthy lifestyle, and improving physical condition
Topics of appearance/weight	Obesity/fat	Reference to preventing or eliminating obesity and burning fat
	Metabolic syndrome/visceral fat	Reference to eliminating visceral fat or improving metabolic syndrome. Metabolic syndrome is included here since it is related to obesity rather than disease prevention in the Japanese context.
	Weight/body shape	Reference to body shape and weight, such as proper weight, weight loss, and firm body
Topics of quality-of-life	Mental health/Stress reduction	Reference to stress reduction, improved mental health, relaxation, and refreshment
	Better sleep/Recovery from fatigue	Reference to better sleep and recovery from fatigue
	Other QOL	Reference to other QOL-related benefits such as improved concentration
Categories of Subordinate Goals	Daily life activities	Activities associated with labor and household tasks necessary for daily living
	Exercise	Activities planned during leisure time with the intention of improving health, physical fitness, or enjoyment
Topics of daily life activities	Daily life mobility	References to ways of increasing physical activity in daily mobility, such as walking or biking to work, school, and shopping
	Stair use	Reference to using stairs instead of using the elevator or escalator
	Household tasks	Reference to performing household tasks such as washing clothes, sweeping floors, and cleaning windows
	Moving frequently	Reference to standing up and moving frequently
	Leisure time life activity	Reference to leisure time life activities, such as gardening, playing with children and walking the dog
Topics of exercise	Walking	Walking with no mention of daily life mobility
	Jogging/Running	Jogging and running
	Cycling	Cycling with no mention of daily life mobility
	Swimming	Swimming and walking in water
	Other aerobic exercises	Dancing, yoga, Tai Chi, mountain climbing, etc.
	Strength training	Resistance training, push-ups, squats, dumbbell exercise, and any exercise named as strength training
	Stretching	Any exercise named as stretching
	Exercising while doing something	Any exercise that is recommended to be done while performing daily activities*When this exercise refers to other exercise names, it counts as both (e.g., stretching while watching TV)
	Radio calisthenics	Radio calisthenics (popular calisthenics in Japan)
	Training for locomotive syndrome	Training for prevention of locomotive syndrome, named “loco tore” in Japanese
	Local calisthenics	Calisthenics developed by local government
	Other exercises	Any other exercises that do not fit into the above, such as conditioning and preparatory exercise
	Exercising	Exercise overall and going to the gym or sports center (not specified)
	Sports	Sports that require physical effort and skills and are performed for pleasure, such as golf, football, and volleyball

## References

[1] World Health Organization Physical Activity. https://www.who.int/news-room/fact-sheets/detail/physical-activity.

[2] World Health Organization WHO Guidelines on Physical Activity and Sedentary Behaviour. https://www.who.int/publications/i/item/9789240015128.

[3] Ikeda N., Saito E., Kondo N., Inoue M., Ikeda S., Satoh T., Wada K., Stickley A., Katanoda K., Mizoue T. (2011). What Has Made the Population of Japan Healthy?. Lancet.

[4] Ministry of Health, Labour and Welfare Physical Activity Guidelines for People Aged 18 to 64 (Active Guide). https://www.mhlw.go.jp/content/000656517.pdf.

[5] Minister of Health, Labour and Welfare A Basic Direction for Comprehensive Implementation of National Health Promotion. https://www.mhlw.go.jp/stf/seisakunitsuite/bunya/kenkou_iryou/kenkou/kenkounippon21.html.

[6] Ministry of Health, Labour and Welfare (2017). National Health and Nutrition Survey. https://www.mhlw.go.jp/content/10904750/000351576.pdf.

[7] Japan Sport Association Press Release Awareness Survey toward Sports and Exercise. https://www.japan-sports.or.jp/news/tabid92.html?itemid=4593.

[8] Moskowitz G.B. (2012). The Representation and Regulation of Goals. Goal Dir. Behav..

[9] Höchli B., Brügger A., Messner C. (2018). How Focusing on Superordinate Goals Motivates Broad, Long-Term Goal Pursuit: A Theoretical Perspective. Front. Psychol..

[10] Ford M.E. (1992). Motivating Humans: Goals, Emotions, and Personal Agency Beliefs.

[11] Locke E.A., Latham G.P. (2002). Building a Practically Useful Theory of Goal Setting and Task Motivation. A 35-Year Odyssey. Am. Psychol..

[12] Höchli B., Brügger A., Messner C. (2020). Making New Year’s Resolutions That Stick: Exploring How Superordinate and Subordinate Goals Motivate Goal Pursuit. Appl. Psychol. Health Well Being.

[13] Seijts G.H., Latham G.P., Tasa K., Latham B.W. (2004). Goal Setting and Goal Orientation: An Integration of Two Different Yet Related Literatures. AMJ.

[14] Brinkman C., Baez S.E., Genoese F., Hoch J.M. (2020). Use of Goal Setting to Enhance Self-Efficacy After Sports-Related Injury: A Critically Appraised Topic. J. Sport Rehabil..

[15] Bandura A. (1998). Health Promotion from the Perspective of Social Cognitive Theory. Psychol. Health.

[16] Ordonez L.D., Schweitzer M.E., Galinsky A.D., Bazerman M.H. (2009). Goals Gone Wild: The Systematic Side Effects of Over-Prescribing Goal Setting. Acad. Manag. Perspect..

[17] Carver C.S., Scheier M.F. (2001). On the Self-Regulation of Behavior.

[18] Kruglanski A.W., Shah J.Y., Fishbach A., Friedman R., Chun W.Y., Sleeth-Keppler D. (2018). A Theory of Goal Systems. The Motivated Mind.

[19] Segar M.L., Eccles J.S., Richardson C.R. (2011). Rebranding Exercise: Closing the Gap between Values and Behavior. Int. J. Behav. Nutr. Phys. Act..

[20] Segar M.L., Updegraff J.A., Zikmund-Fisher B.J., Richardson C.R. (2012). Physical Activity Advertisements That Feature Daily Well-Being Improve Autonomy and Body Image in Overweight Women but Not Men. J. Obes..

[21] Segar M.L., Eccles J.S., Peck S.C., Richardson C.R. (2007). Midlife Women’s Physical Activity Goals: Sociocultural Influences and Effects on Behavioral Regulation. Sex Roles.

[22] Crawford R. (1980). Healthism and the Medicalization of Everyday Life. Int. J. Health Serv..

[23] Smith D.H., Gravelle H. (2001). The Practice of Discounting in Economic Evaluations of Healthcare Interventions. Int. J. Technol. Assess. Health Care.

[24] Papies E.K. (2016). Goal Priming as a Situated Intervention Tool. Curr. Opin. Psychol..

[25] Weingarten E., Chen Q., McAdams M., Yi J., Hepler J., Albarracín D. (2016). From Primed Concepts to Action: A Meta-Analysis of the Behavioral Effects of Incidentally Presented Words. Psychol. Bull..

[26] Marshall A.L., Leslie E.R., Bauman A.E., Marcus B.H., Owen N. (2003). Print versus Website Physical Activity Programs: A Randomized Trial. Am. J. Prev. Med..

[27] Marks J.T., Campbell M.K., Ward D.S., Ribisl K.M., Wildemuth B.M., Symons M.J. (2006). A Comparison of Web and Print Media for Physical Activity Promotion among Adolescent Girls. J. Adolesc. Health.

[28] Elliott L.R., White M.P., Taylor A.H., Abraham C. (2018). How Do Brochures Encourage Walking in Natural Environments in the UK? A Content Analysis. Health Promot. Int..

[29] Gainforth H.L., Barg C.J., Latimer A.E., Schmid K.L., O’Malley D., Salovey P. (2011). An Investigation of the Theoretical Content of Physical Activity Brochures. Psychol. Sport Exerc..

[30] Ministry of Health, Labour and Welfare. https://www.mhlw.go.jp/index.html.

[31] e-Health Net. https://www.e-healthnet.mhlw.go.jp/information/exercise.

[32] Smart Life Project. https://www.smartlife.mhlw.go.jp/.

[33] Japan Sports Agency. https://www.mext.go.jp/sports/.

[34] StatCounter Search Engine Market Share Japan. https://gs.statcounter.com/search-engine-market-share/all/japan.

[35] Ministry of Health, Labour and Welfare (2013). Physical Activity Reference for Health Promotion. https://www.mhlw.go.jp/stf/houdou/2r9852000002xple.html.

[36] Ministry of Health, Labour and Welfare Specific Health Checkups and Specific Health Guidance. https://www.mhlw.go.jp/stf/seisakunitsuite/bunya/kenkou_iryou/kenkou/seikatsu/index.html.

[37] Landis J.R., Koch G.G. (1977). The Measurement of Observer Agreement for Categorical Data. Biometrics.

[38] Vohs K.D., Kaikati A.M., Kerkhof P., Schmeichel B.J., Moskowitz G.B., Grant H. (2009). Self-Regulatory Resource Depletion: A Model for Understanding the Limited Nature of Goal Pursuit. Psychology of Goals.

[39] Chun W.Y., Kruglanski A.W., Sleeth-Keppler D., Friedman R.S. (2011). Multifinality in Implicit Choice. J. Pers. Soc. Psychol..

[40] Grabe S., Ward L.M., Hyde J.S. (2008). The Role of the Media in Body Image Concerns among Women: A Meta-Analysis of Experimental and Correlational Studies. Psychol. Bull..

[41] Danthinne E.S., Giorgianni F.E., Rodgers R.F. (2020). Labels to Prevent the Detrimental Effects of Media on Body Image: A Systematic Review and Meta-Analysis. Int. J. Eat. Disord..

[42] Spettigue W., Henderson K.A. (2004). Eating Disorders and the Role of the Media. Can. Child Adolesc. Psychiatr. Rev..

[43] Wang Y., Li P., Zhang B., Han Y. (2022). Does Cognitive Attitude Matter When Affective Attitude Is Negative in Physical Activity Behavior Change?. Res. Q. Exerc. Sport.

[44] Chaabane S., Chaabna K., Doraiswamy S., Mamtani R., Cheema S. (2021). Barriers and Facilitators Associated with Physical Activity in the Middle East and North Africa Region: A Systematic Overview. Int. J. Environ. Res. Public Health.

[45] Baert V., Gorus E., Mets T., Geerts C., Bautmans I. (2011). Motivators and Barriers for Physical Activity in the Oldest Old: A Systematic Review. Ageing Res. Rev..

[46] Joseph R.P., Ainsworth B.E., Keller C., Dodgson J.E. (2015). Barriers to Physical Activity Among African American Women: An Integrative Review of the Literature. Women Health.

[47] Japan Health Promotion & Fitness Foundation (2018). National Local Calisthenics Fact-Finding Survey. https://www.health-net.or.jp/tyousa/houkoku/gotouchi.html.

[48] Ishihara M., Takiguchi T., Ishigami K. (2019). Factors Associated with the Lack of Implementation of Local Gymnastics among Rural Community-Dwelling Elderly. Niigata J. Health Welf..

